# Chinese Medicine: a peer-reviewed open access journal for evidence-led Chinese medicine

**DOI:** 10.1186/1749-8546-1-1

**Published:** 2006-11-23

**Authors:** Hin Wing Yeung

**Affiliations:** 1 International Society for Chinese Medicine (ISCM), A & C, 1^st ^Floor, Block 2, University of Macau, Av. Padre Tomas Pereira, Taipa, Macao, China

## What is Chinese Medicine?

The new journal, Chinese Medicine (CM), is a peer-reviewed, open access, international, interdisciplinary and scholarly journal in Chinese medicine. CM aims to provide evidence-led force for the advancement of Chinese medicine research. CM is the official journal of the International Society for Chinese Medicine (ISCM) [1] with academic support from leading research institutes as its members and financial support from the Macao Foundation of the Macao Special Administrative Region, China. Chinese Medicine will serve as an unbiased and non-commercial platform for publishing advanced Chinese medicine research. An open access online version of CM is published by BioMed Central (BMC) [2], a London-based leading publisher of biomedical journals. ISCM also publishes a print version of CM.

CM publishes articles reporting valuable research with general interest in any aspect of Chinese medicine, such as basic research, clinical research, methodologies, theories and diagnoses, *materia medica*, acupuncture and other non-pharmacologic modalities of the Chinese medical system. CM welcomes submission of papers on application of Chinese medicine in disease prevention, treatment/management, and rehabilitation. In addition to biomedical research, CM also accepts reports on various aspects of Chinese medicine research, including public health, regulation, management, education, technical translation, cultural exchange and internationalization. Multidisciplinary and translational research reports are also strongly encouraged. All research and review articles must be peer-reviewed to ensure that the valuable knowledge drawn from them is evidence-based, scientifically justified and ethical. Peer-review is conducted by at least two internationally recognized experts in the respective fields. Our high calibre reviewers and editors will only accept papers of high quality.

## Why a new Chinese medicine journal?

### To meet the demand

Since the 1990s, there has been a phenomenal growth of interest in traditional medicine knowledge and practices, which is evident in the increasing World Health Organization (WHO) activities and publications, such as Global Strategy for Traditional Medicine (2002–2005) [3]. Research activities in Chinese medicine, one of the most used and best documented traditional medicines, have been increasing tremendously worldwide as well. A large portion of the research in Chinese medicine has been carried out in Asia, particularly in China, Japan and Korea, and reported in their native languages. As a result, currently available Chinese medicine literature published in English language journals does not meet the growing demand from the international scientific community for easy access to research findings in this field. Moreover, most Chinese medicine literature is scattered throughout a wide range of scientific journals of which Chinese medicine is often not the main concern. This also hinders easy access to Chinese medicine research. CM aims to fill this gap and tackle the problems.

### To advocate evidence-led Chinese medicine (ELCM)

Our concept of evidence-led Chinese medicine (ELCM) is broader than that of evidence-based Chinese medicine (EBCM). We believe that the advancement, including modernization and internationalization, of Chinese medicine should be led by evidence. Chinese medicine research should gather new evidence which can be used to break new grounds and/or to verify old traditions. For fruitful research, ELCM should be: (1) inheriting without dogmatic restriction; (2) innovating without random deviation; and (3) reasoning for true understanding. Similar opinion was echoed in the consensus reached at the 2004 ISCM International Expert Forum [4]. ELCM treats all appropriate research methodologies and evidences equally, irrespective of whether they are traditional or modern, conventional or alternative, macroscopic (systems and/or subsystems) or microscopic (cellular and/or molecular) as long as scientific rigour is upheld.

Chinese medicine is a treasure of the Chinese people, who not only have been benefiting from its well-known clinical effectiveness but also have been inspired by its ancient wisdom. With a natural and holistic approach, in addition to disease treatment, Chinese medicine does care about a person's well-being. It restores and maintains the dynamic balance of a person, and achieves a harmony between human and nature. While Chinese medicine is based on a holistic system that is inherently different from the modern biomedical sciences, its holistic theories and practices are not contradictory to general principles of logic and science. Chinese medicine sees all components of the whole person (system) interact dynamically with one another to maintain a balanced physiological state. Serious imbalance results in a diseased state. Conventional investigations on isolated components and/or processes are missing the big picture and therefore will not fully unlock the scientific value of Chinese medicine. The situation had remained unchanged until systems biology, omics and related technologies, new approaches and methodologies for scientific study of Chinese medicine began to emerge. From the perspective of systems biology and omics, all components in a living organism/system interact dynamically to maintain homeostasis (i.e. balance or equilibrium), and serious deviations from homeostasis cause diseases. This idea is in line with the holistic concept and practices of Chinese medicine. Chinese medicine research adopting the approaches and methodologies of systems biology and omics as well as those from other relevant fields, such as evidence-based medicine (EBM) and clinical and molecular epidemiology, will generate macroscopic, microscopic and integrative macroscopic-microscopic evidences for ELCM. Supported by an international community of active researchers in the relevant fields, CM will advocate ELCM in order to help unlock the scientific value of Chinese medicine.

## Why submit your manuscript to Chinese Medicine?

To better serve its authors and readers, CM strives to ensure that the best Chinese medicine research is published, and read by the widest possible range of international audience. Open access journals provide the best solution. While it is true that most of the established and printed journals are available online, access to an article usually requires a journal subscription or a purchase of an individual article. Due to the high costs of subscription fees, many research groups or libraries around the world, especially those in the developing countries, are cutting down on the numbers of subscribed journals. This limits the dissemination of research findings.

Supported by strong Editorial and Advisory Boards, and working closely with BioMed Central, an open access publisher based in London, we make all articles published in the journal totally free to anyone who has access to the Internet. Moreover, unlike many other journals within the BMC family where authors pay an article processing charge (APC), CM pays the APC for all authors in full, so that authors have no financial burden to publish articles.

Our authors retain the copyright for their work and are free to email the articles to their colleagues, post them on the Internet, grant anyone the right to reproduce and disseminate them, provided that it is correctly cited and no errors are introduced. Apart from being available online, articles published in our journal are also archived permanently in PubMed Central [5], the US National Library of Medicine's repository of life science literature, and in repositories at the University of Potsdam [6] in Germany, at INIST [7] in France and in e-Depot [8], the National Library of the Netherlands' digital archive of all electronic publications. This means that articles published in CM have high international visibility and accessibility, and are tremendously citation friendly. Studies have also provided strong evidence that open access (OA) articles are more immediately recognized and cited by peers, leading to a quicker and higher number of citations [9-11].

The distinctive features of CM, provided by BioMed Central and our editorial office, offer special advantages to our authors, readers, the scientific and medical communities and the general public. The efficient electronic publishing process sustained by BioMed Central ensures easy online submission and fast online peer review and publication. Furthermore, there are no length restraints or figure limits and large datasets are also accepted. Articles are published immediately upon acceptance and soon after indexed by PubMed and other major repositories, meaning that they will be read and searched quickly and globally. Our editorial office believes that the more important the submitted articles (i.e. papers of great interest and high profile), the more they deserve the best treatment: (1) fast, thorough, fair peer reviews by the most appropriate referees; (2) effective presentation and professional formatting of the articles both on the web and in print; and (3) promotion of the articles to all interested parties. In a speedy and professional manner, the scientific value and potential impact of all published articles in CM will be judged and evaluated by the international scientific community.

## Conclusion

Chinese Medicine is an exciting medium for fostering evidence-led force for the advancement of Chinese medicine by committing to professional excellence and open access publishing. CM aims to promote the generation of a research knowledge base in Chinese medicine. CM is devoted to bridging the gap between traditional Chinese medicine and modern medicine. CM will endeavour to unlock the scientific value of Chinese medicine. We believe that it is now high time for a renaissance of traditional Chinese medicine and that the success of this journal relies very much on its editors working in concert with its reviewers and authors to keep publishing high quality papers in ELCM. Please join us and help make this happen.
